# Knowledge gaps in the construction of rural healthy homes: A research agenda for improved low-cost housing in hot-humid Africa

**DOI:** 10.1371/journal.pmed.1002909

**Published:** 2019-10-10

**Authors:** Lorenz von Seidlein, Hannah Wood, Otis Sloan Brittain, Lucy Tusting, Alexa Bednarz, Salum Mshamu, Catherine Kahabuka, Jacqueline Deen, David Bell, Steve W. Lindsay, Jakob Knudsen

**Affiliations:** 1 Mahidol Oxford Tropical Medicine Research Unit, Faculty of Tropical Medicine, Mahidol University, Bangkok, Thailand; 2 Centre for Tropical Medicine and Global Health, Nuffield Department of Medicine, University of Oxford, United Kingdom; 3 Ingvartsen Architects, Copenhagen K, Denmark; 4 Department of Disease Control, London School of Hygiene & Tropical Medicine, London, United Kingdom; 5 Bill and Melinda Gates Foundation, Seattle, Washington, United States of America; 6 CSK Research Solutions Ltd., Dar es Salaam, Tanzania; 7 University of the Philippines, Manila, Philippines; 8 Independent Consultant, Issaquah, Washington, United States of America; 9 Department of Biosciences, Durham University, Durham, United Kingdom; 10 School of Architecture, The Royal Danish Academy of Fine Arts, Copenhagen, Denmark

## Abstract

Lorenz von Seidlein and colleagues discuss improving house designs in rural Africa to benefit health.

**Summary points**

- The population of Africa is projected to increase 3-fold before 2100. Enormous resources will be required to construct houses for the predicted additional 2.5 billion new inhabitants (alongside provision of food, education, medical care, and other essentials).
- This population growth presents an opportunity to incorporate housing concepts that can improve health and well-being.
- Well-designed houses should provide thermal comfort and a barrier to separate disease-carrying vectors from residents, decrease indoor pollution, and incorporate water supply, sanitation, electricity, and security.
- We identified the following research priority areas: optimal building shape, roofing, vector barriers, cladding, water supply, sanitation, kitchen design, electricity supply, rural to periurban housing, and user acceptability.
- Improved housing incorporating novel design elements tailored to local environments in the hot-humid regions of Africa will require investments that could come through innovative financing mechanisms for residential property. Better housing will promote well-being and health and play a critical role in achieving the Sustainable Development Goals (SDGs).

## Introduction

According to the United Nations’ medium-scenario projections, Africa’s population will rise to 2.5 billion in 2050 and to more than 4 billion in 2100, which is the fastest population growth rate in the world [1]. This population growth in tropical Africa results in a large and unmet demand for appropriate housing, which contributes to insecurity, poor health, and migration. Nevertheless, sub-Saharan Africa is predicted to have the second fastest growing regional economy by 2020 [2], which would be an opportunity to invest in healthy house designs that help meet targets on disease and well-being [3]. There is a notion that the rural population in Africa is stable or even shrinking due to urban migration [4]. In fact, the rural population is increasing rapidly (1.9% annual population growth in 2016) albeit at a slower rate than the urban population (4.1% annual population growth in 2016) [5]. With the steady proliferation of cheap public transport by bus and motorbike, the rural population has become more mobile [6], allowing rural residents to seek work elsewhere while maintaining (and possibly improving) their village residence.

**Fig 1:**
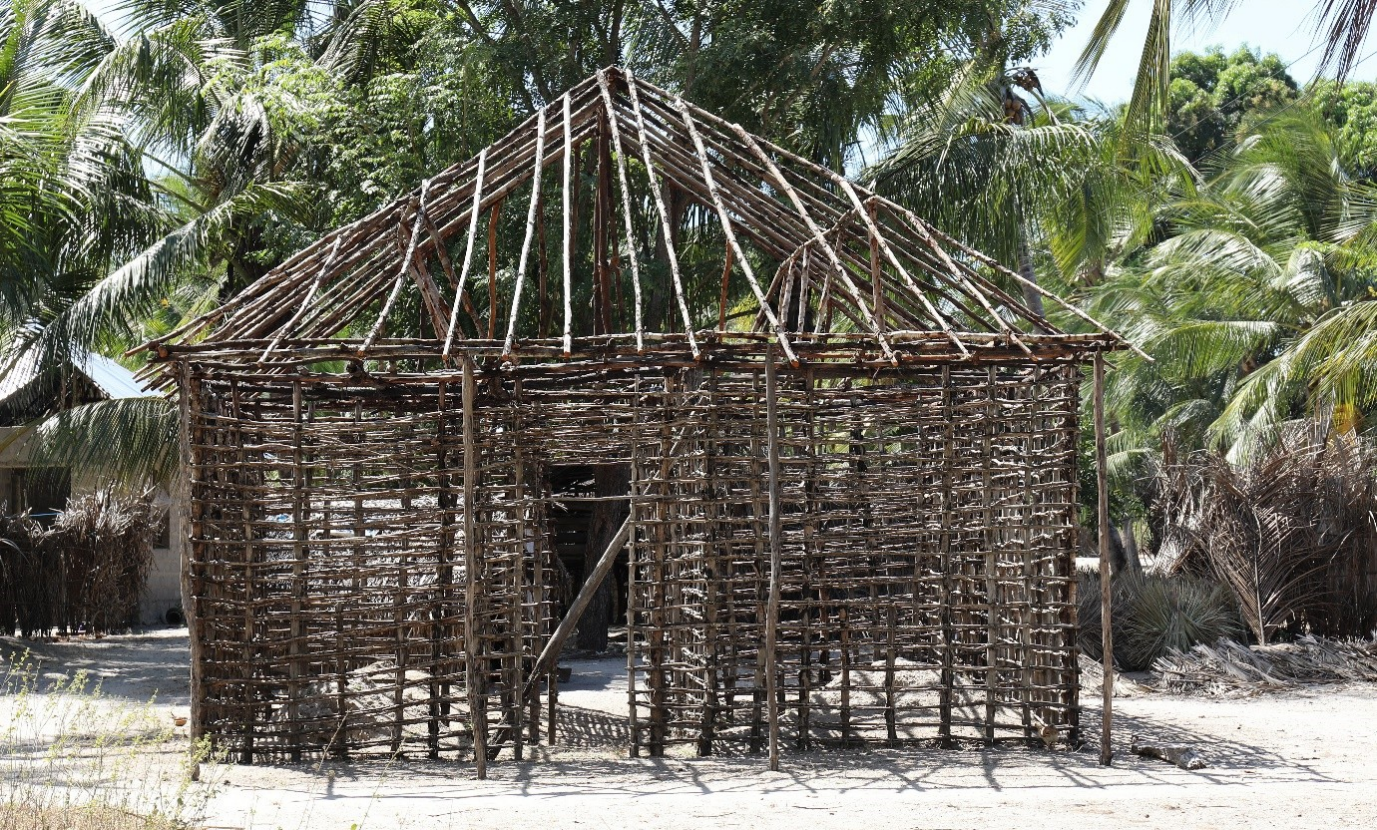
Traditional mud hut (wattle and daub) construction seen in Mtwara, Tanzania. Once the woven lattice of sticks (wattle) is completed, the gaps are filled and plastered (daubed) with sticky mud and stones (photo by Lorenz von Seidlein).

**Fig 2:**
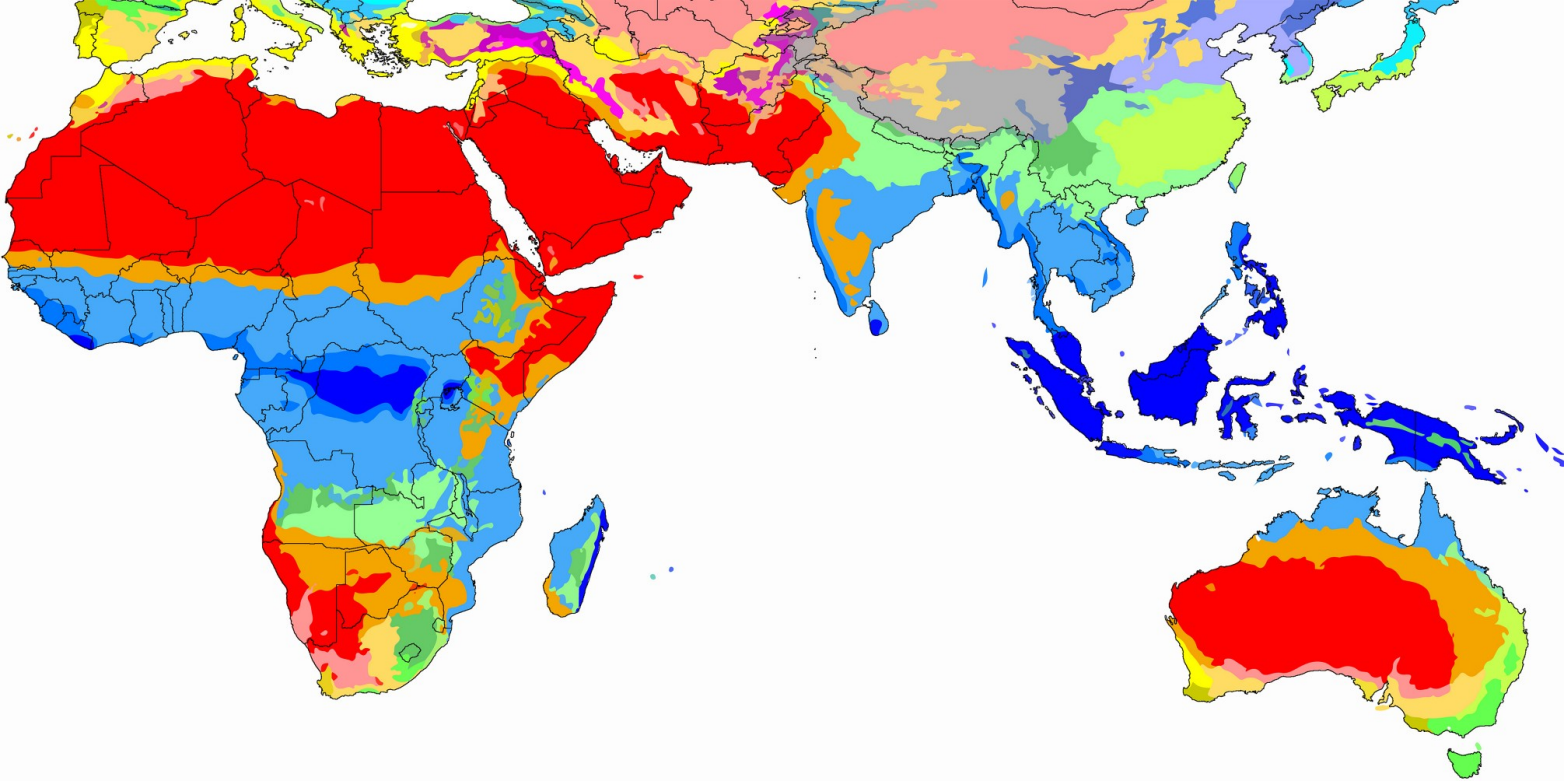
Koeppen climate classification (blue = hot, humid, tropical; red = hot, dry, arid; and green = temperate) [9].

The mud house, a basic wattle and daub construction with a thatched roof, is the most common housing type throughout rural Africa (Fig 1) [7]. While wattle and daub is increasingly replaced by burnt brick and concrete and thatched roofs are replaced with metal sheets, the overall house design, including the pounded earth floor, remains unchanged [8]. Heavy, poorly ventilated single story buildings are the norm. The resultant design with its thick walls and heavy roofs provides a high thermal mass that can provide comfort in arid African climates (Fig 2), where temperatures are high during the day but drop at night. Heat is absorbed by the thick walls and floors during the day, then radiates into the house during cold nights. By early morning, the building has cooled down and trapped cool air, and cooled walls stabilize temperature as the outside temperature rises. Radically different building designs are needed for thermal comfort in hot-humid climate zones, typical of much of Africa (Fig 2, blue zones). In this region, temperatures remain high during the day and night, although climate may differ by altitude and proximity to large bodies of water. Tropical Asian countries with a similar hot-humid climate have traditional house designs that are much different from the African mud house [7]. The traditional South East Asian house is a light construction with a minimal thermal mass, frequently elevated on stilts, with porous cladding that optimizes airflow through walls and the building.

Well-constructed buildings can prevent the entry of mosquitoes—the vector for a range of infections, including malaria and dengue fever [8]—and flies, which transmit enteric pathogens and spread *Chlamydia trachomatis*, the organism causing trachoma that can lead to blindness [10–12]. Similarly, improved design and construction of homes can address risk factors to health, including indoor pollution from cooking smoke, which is a major risk factor for acute and chronic respiratory tract diseases [13–18]. Mold can trigger allergic respiratory diseases [19,20], and radon—a radioactive gas derived from rock and soil—is associated with an increased cancer risk [21]. The absence of a hygienic kitchen, safe water supply, and adequate sanitation predisposes inhabitants to enteric diseases. Replacing dirt floors with concrete significantly improves the health and cognitive development of young children by decreasing diarrhea, parasitic infestations, and anemia [22]. Besides the direct consequences, poor housing has additional, less tangible but still harmful, effects. Without an illuminated indoor space, children are unable to study outside daylight hours [23]. Overcrowding and inadequate ventilation interfere with sleep and limit productivity, growth, and psychological well-being [24]. As Africans upgrade or build new dwellings to improve their living conditions, they could incorporate design features that protect against illness and increase comfort. This paper shows a range of possibilities for improving rural housing in hot-humid Africa [7,25] but also discusses knowledge gaps regarding optimal designs and structures.

## Areas for research and improvement

We discuss here urgent research questions in the construction of rural healthy homes in a thematic order (Table 1).

**Table 1:** Research questions for healthy homes in hot-humid tropical Africa.

1. The basic building shape	How can CFD help us to improve the geometry of rural homes? Which features of a house are important to reduce mosquito entry? What is the minimum/optimal house elevation to minimize mosquito entry?
2. Roofing	Which roofing materials support water harvesting and support indoor thermal comfort? How can solar power be incorporated into roofs while maintaining indoor comfort?
3. Vector barriers (bednets, screens, windows, and doors)	Can long-lasting residual insecticides be integrated in high-performance polyethylenes? Which fibers are optimal for screening of the house? What is the optimal screening material? Can we improve window and door designs to make them more user friendly, e.g., self-closing?
4. Cladding	Which cladding materials allow optimal ventilation while fulfilling safety, privacy, and aesthetic expectations?
5. Water supply and sanitation	What is the most cost-efficient approach to provide safe water supply all year round? What is the optimal latrine and hand-cleaning station design and location in relation to the home?
6. Kitchen design	How to design a user-friendly fireplace that minimizes indoor pollution while being safe and acceptable for users? Which chimney design could be prefabricated? How to promote efficient, smokeless cook stoves that burn biogases or LPG?
7. Electricity supply	Can solar power be harvested in an appropriate, affordable fashion in hot-humid, rural Africa? Can solar energy be stored in an affordable fashion?
8. Urbanization	Which elements in novel rural homes could be applied to periurban homes?
9. Acceptability: the user perspective	How can design innovations, including elevation, novel roofing materials, windows, and doors, benefit the largest possible number of end users?
10. Collaborations	How to interest local architects to collaborate in the design and construction of low-cost rural homes? How to train local craftsmen in the use of new building techniques and unfamiliar construction materials?
11. Economics and financing	Which design innovations are cost-effective and how can they be funded? What is the best way to organize loans for low-cost housing in rural Africa?
12. Maintenance	How should we instill a maintenance culture for improved houses so that they last longer and have meaningful health benefits?

**Abbreviations:** CFD, computational fluid dynamics; LPG, liquefied petroleum gas

### 1. The basic building shape can fundamentally influence airflow and indoor climate

**Fig 3:**
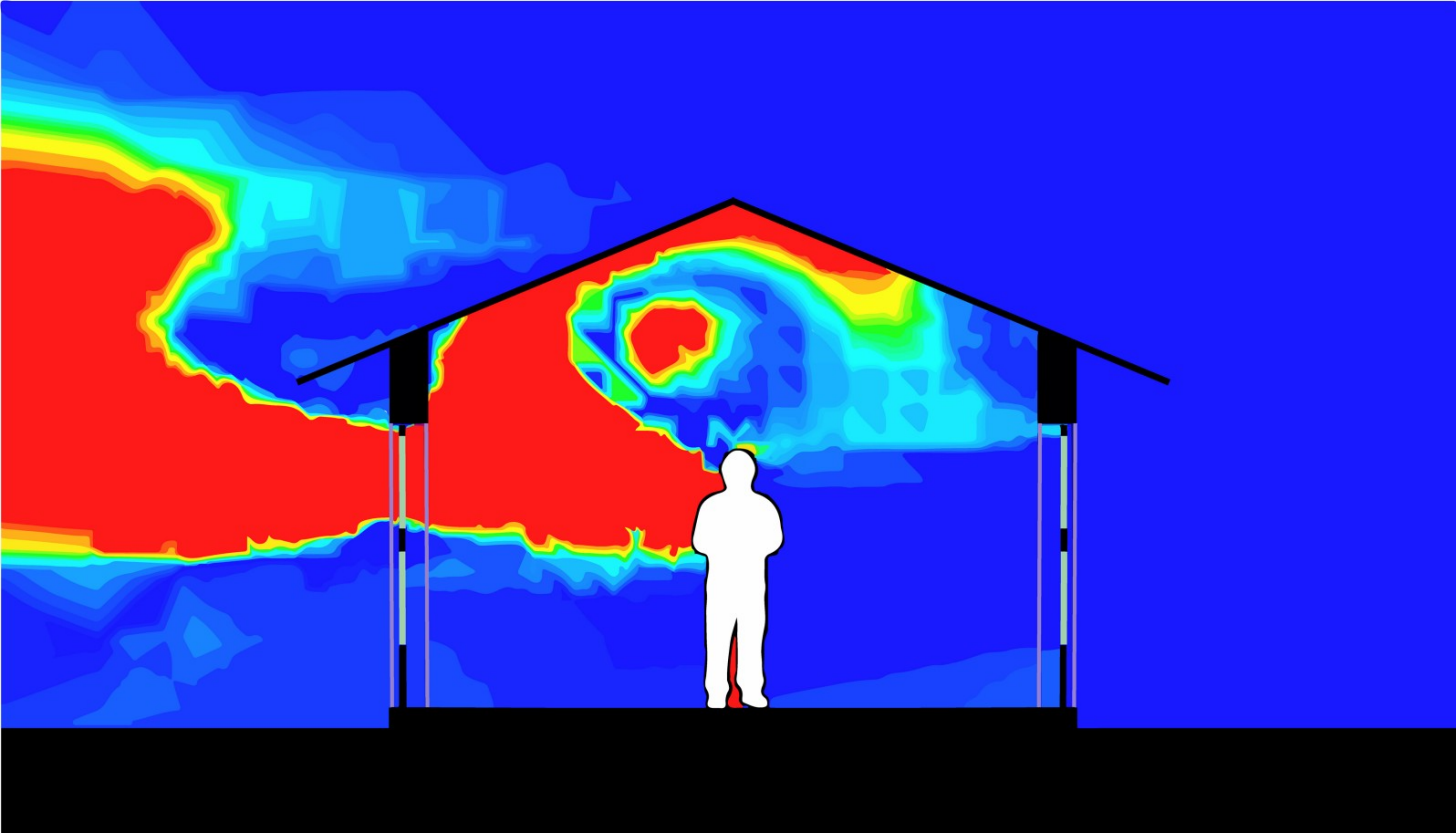
Using CFD to visualise CO_2_ concentrations inside a house covered by a corrugate iron roof in The Gambia. CO_2_ escapes mostly through a screened opening in the door. Red represents highest and blue the lowest concentrations of CO_2_. CFD is increasingly used to optimise the indoor climate in the design of new buildings. CFD, computational fluid dynamics.

Increased airflow leading to improved thermal comfort could encourage the use of bednets and reduce the risk for malaria. Simulating the mixture, temperature, and movement of CO_2_ and other gases is mathematically challenging but has become more accessible through computational fluid dynamics (CFD) software, such as Autodesk CFD (Autodesk, Mill Valley, CA; Fig 3). CFD software can simulate temperature, humidity, wind speed, and the dispersion of CO_2_, a critical part of the human odour plume that attracts mosquitoes, including *Anopheles*, the vector for *Plasmodium* and other infections. For example, elevating homes may be beneficial in reducing mosquito entry, yet by how much the house should be elevated remains an open question. Increasing the height to improve airflow increases costs, but does it result in further reductions of mosquito density? Here, modelling is of little help as the behavior of the mosquitoes is not well understood [26,27]. Entomological studies are needed to provide critical information.

### 2. Rural communities are shifting from thatch to metal sheet roofs [28]

Thatched roofs release dust and small fragments of dirt that reduce indoor air quality; provide a hiding place for vermin, snakes, and insects; become highly flammable during the dry season; and need to be replaced regularly. Metal roofs can be changed less frequently but provide less thermal insulation, produce excessive noise during heavy rainfall, and can be dangerous when detached by high wind speeds. There is a need to assess the cost-effectiveness of alternative roofing material prototypes, for example, composite materials such as laminated bamboo [29], cement-based composites, and recycled plastic [30] in varying climatic zones [31]. Collaborations with material researchers and producers to provide a new generation of affordable roofing materials are of a high priority. Critically, to be accepted, the functionality of new roofing materials must be balanced with aesthetic considerations.

### 3. In traditional African mud houses with closed eaves and without windows, the door is the only entry point for mosquitoes

Novel door designs aim to exclude mosquitoes while allowing ventilation and entry of light and maintaining privacy. Keeping the door shut is critical to exclude insects, therefore a spring mechanism to keep doors shut is important. Similarly, windows should be light, affordable, air permeable, and prevent mosquito entry. Safety is critical in preventing entry of burglars while allowing residents to escape in case of fire. Prefabricated mosquito-proof doors and windows can provide these features at a high quality and reduced cost. The ideal screening material is affordable, robust, replaceable, permeable to air and light but not insects, and provides a sense of security. Shade nets, an underutilized but practical and affordable material, can be used for cladding and screening. The use of novel synthetic materials such as Aramid fibers and high-performance polyethylenes deserves to be explored as screening materials. More empirical evidence is needed to understand how different materials perform as screens and nets, considering acceptability, practicality, costs of mass production, recycling, and eventual disposal.

### 4. Cladding, the skin of the building, should be air permeable

While mud or burned brick may be the most familiar cladding materials in Africa, they interrupt airflow, are heavy, costly, and therefore not ideal in hot-humid climates. Loosely spaced timber planks allow some airflow but are expensive and vulnerable to damage from termites unless treated. Bamboo is a preferred cladding material in rural South East Asia [32] but is underutilized in most of Africa, despite bamboo growing naturally in the region. Currently, there is limited expertise in farming and treating bamboo in Africa despite a growing market. Mats of woven palm leaves are used in Asia as cladding material, e.g., Nipa huts in the Philippines, but are less frequently seen in Africa. Shade nets started as roofing material in greenhouses but can be used as cladding material and will become cheaper with increasing demand. Shade nets have considerable potential in construction particularly when incorporated with other materials (e.g., bamboo slats) that provide the rigid structure.

### 5. There is a broad consensus that unsafe water and poor sanitation predispose to gastro-enteric infections and increase the risk of trachoma

Currently in many villages in tropical Africa, rainwater is collected and stored in buckets; otherwise, water must be carried from the nearest source. Better alternatives include boreholes, covered communal wells, or rainwater harvesting, with suitability depending on rainfall patterns and depth and purity of ground water. Rainwater collection and storage are used in many regions and require only minimal engineering: a gutter, piping, a basic water filtration system, and a water container. For collection, the roof surfaces should be large, clean, and have an appropriate angle to prevent water accumulation. The gutter and fixing system must be sufficiently robust to cope with torrential rainfall, a perennial challenge in tropical climate regions. First, flush water diverter systems can prevent the most contaminated rainwater from entering the tank and are a cost-effective and low-tech addition to a rainwater filtering system [33]. Water needs to be stored in a way to prevent mosquitoes breeding in the containers. Open defecation remains a challenge in low-income countries [34], potentially contaminating water sources. Provision of latrines that prevent fly breeding, eliminate odor, have running water, are easily cleanable, are illuminated at night, and are children friendly would be ideal. Handwashing with soap is a cost-effective measure for reducing the transmission of diarrhoeal diseases, yet many latrines lack a handwashing facility and, most importantly, sufficient water [35].

### 6. Kitchens should be designed to allow easy access to an appropriate food preparation area with hygienic surfaces that prevent cross-contamination between fresh and cooked food

Barriers against rodents and flies are critical to maintain sanitation and prevent illness. Less obvious but equally important is the need for practical, cost-effective cooking facilities. The design of chimneys can be optimized to draw smoke effectively out of the house using CFD as part of an iterative design process. Optimized chimneys could be prefabricated, saving time and money. The use of high-efficiency wood and biofuel stoves will greatly reduce emissions while also reducing fuel consumption and deforestation [36,37]. Reducing deforestation for cooking is essential in view of projected population growth. The long-term solution will be a switch to cleaner fuels such as ethanol, biogas, or liquefied petroleum gas [38].

### 7. In 2016, only 43% of all households in Africa had access to a stable power source [39]

Perhaps the most promising source of electricity in rural Africa is solar energy. Small affordable solar panels sufficient to power light sources and recharge phones and water pumps are becoming commonplace in parts of rural Africa. Access to electricity from solar panels during daytime is sufficient to charge phones or power a water pump and may even run a ceiling fan. Uninterrupted access to smartphones is increasingly important for commerce, healthcare, and social interaction. Solar panels have become cost-effective, but energy storage remains problematic.

### 8. The increased numbers of people seeking work in cities that has led to the rapid growth of improvised, informal periurban and urban zones without the necessary supporting infrastructure is one of the major global challenges of our time [40,41]

Some housing issues are shared by rural, periurban, and urban populations, but for many issues, different approaches are needed in urban as compared with rural areas. Addressing the broad range of problems encountered in urban slums requires focused research. Applying collective knowledge from previous studies, urban planning insights, and expertise from tropical cities around the world will aid the development of a strategy for healthy homes suitable for dense, urban settings.

### 9. While affordability, supply, and perception of privacy and security are key factors when deciding on house design and materials, appearance and cultural acceptance are critical for success

It is essential to involve and listen to the client to understand what house designs are desirable and what is unacceptable. Many aspects of novel house designs contravene traditional rural house designs; for example, elevating houses and adding a second floor are not common practice in rural tropical Africa. Wider social factors may also need to be addressed: for example, the absence of mud or brick walls could make residents feel unprotected. Understanding which modifications have the highest priority and what is difficult to accept is crucial for success.

### 10. To make a sustained impact in improved housing, there must be collaborations with local architects, schools of architecture, and craftsmen

Because the large and financially most rewarding building projects tend to cluster in the urban environment, incentives are needed to interest teaching institutions and younger architects in the design of low-cost housing for a rural environment. Innovative constructions offer local craftsmen opportunities to acquire new, marketable skills and opportunities to introduce novel materials.

### 11. Funding agencies are typically focused on disease-specific interventions, while more global interventions such as improved housing are unfamiliar and therefore not funded

To interest donors, evidence of affordability, benefits, and costs compared to current housing is critical. This requires both accurate costing of novel housing and the precise assessment of the prevented morbidity and mortality. When these endpoints are not obtainable from empirical field studies, mathematical modelling can play a role.

### 12. Finally, for sustained benefit, improved housing must be well maintained

Maintenance of the houses can be challenging for the rural community where maintenance may have a low priority. How to maintain housing in the rural communities in Africa is a large and often overlooked challenge.

## Conclusions

**Fig 4:**
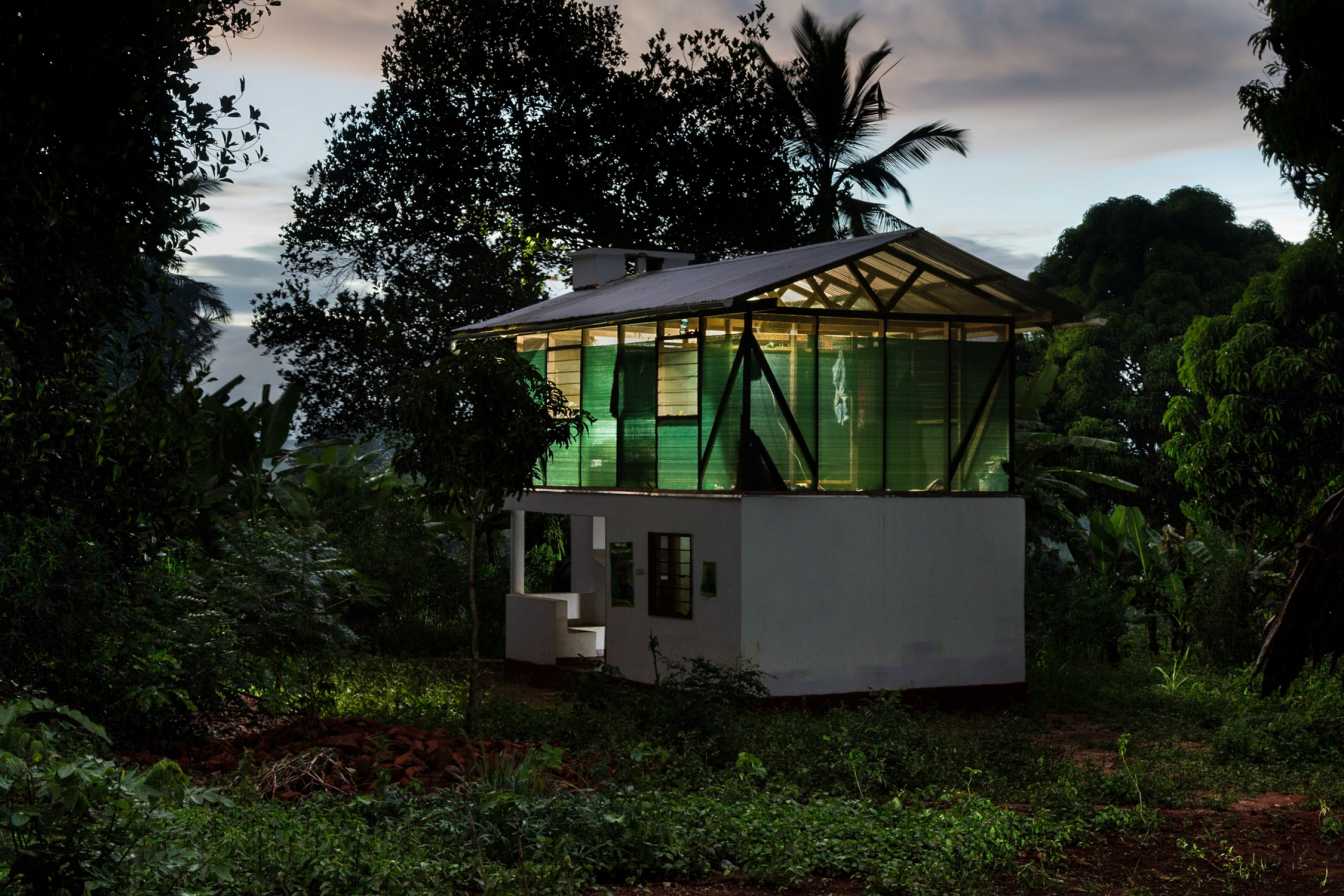
An example of novel-design, double-story building in Magoda, Tanzania. Indoor illumination illustrates the light- and air-permeable character of shade net cladding (photo by Konstantin Ikonomidis).

There are several important research questions in the provision of more appropriate housing in hot-humid, rural Africa (Table 1). We hope that more researchers, particularly from institutions within the African region, will take up this challenge and explore new approaches to fill the increasing demand for housing in Africa. Constructing a new house based on novel designs required between USD $4,231 and $6,220 in a pilot study in Tanzania, substantially larger investments than are required for currently implemented interventions such as insecticide-treated bednets [25]. Novel loan mechanisms for low-cost housing that do not qualify for traditional bank loans and mortgages are under development. Leveraging mechanisms set up to accelerate progress towards key Sustainable Development Goals (SDGs), including good health and well-being (Goal 3), clean water and sanitation (Goal 6), and sustainable cities and communities (Goal 11), could be an important mechanism. Finding appropriate ways to fund, or partially fund, homes to encourage stewardship and self-reliance is a major challenge [42]. Improvements in many aspects of housing described here can be sequential (Fig 4). Once a modification has been successfully introduced and local capacity established, other villagers may start to imitate this feature. For example, the use of bamboo as a cladding material was adopted by local craftsmen in Magoda, a village in Tanzania, after this material was used in a study [25]. Social media have become omnipresent in rural Africa and can be employed to showcase novel developments to larger populations [43]. It may be counterproductive and inappropriate to introduce the same new house design throughout a region, not to mention one as diverse as hot-humid Africa. There is a need to customize designs according to climate variations that are determined by latitude and altitude. The key may be to have sets of solutions for given environments, which are driven by an understanding of individual needs and comfort.

## Abbreviations

CFD: computational fluid dynamics
SDG: Sustainable Development Goal
